# WAVE2 is regulated by multiple phosphorylation events within its VCA domain

**DOI:** 10.1002/cm.20323

**Published:** 2009-01

**Authors:** Shirin M Pocha, Giles O Cory

**Affiliations:** Department of Biochemistry, Faculty of Medical and Veterinary Sciences, University of BristolBristol, United Kingdom

**Keywords:** actin, Arp2/3 complex, CK2, cytoskeleton, ruffles

## Abstract

The (Wiskott-Aldrich Syndrome Protein)-family verprolin homologous protein (WAVE) family of proteins occupies a pivotal position in the cell, converting extracellular signals into the formation of branched filamentous (F) actin structures. WAVE proteins contain a verprolin central acidic (VCA) domain at their C-terminus, responsible for binding to and activating the Arp2/3 complex, which in-turn nucleates the formation of new actin filaments. Here we identify five Casein Kinase 2 (CK2) phosphorylation sites within the VCA domain of WAVE2, serines 482, 484, 488, 489, and 497. Phosphorylation of these sites is required for a high affinity interaction with the Arp2/3 complex. Phosphorylation of ser 482 and 484 specifically inhibits the activation of the Arp2/3 complex by the WAVE2 VCA domain, but has no effect on the affinity for the Arp2/3 complex when the other phosphorylation sites are occupied. We demonstrate phosphorylation of all five sites on endogenous WAVE2 and show that their mutation to non-phosphorylatable alanine residues inhibits WAVE2 function in vivo, inhibiting cell ruffling and disrupting the integrity of the leading edge of migrating cells. Cell Motil. Cytoskeleton 2008. © 2008 Wiley-Liss, Inc.

## INTRODUCTION

The regulated polymerization of actin monomers into filaments underlies several cellular processes including the protrusion of cell membranes, endocytosis and the establishment of polarity [Ridley, 2006]. The formation of new actin filaments is energetically unfavourable under cellular conditions. This enables the tight regulation of actin dynamics via a number of catalytic factors which allow for the spatial and temporal control of Factin production [Pollard, 2007]. The first of these factors identified was the Arp2/3 complex, which stimulates the formation of branched actin structures seen in the leading edge lamellipodia of migrating cells [Machesky et al., 1999].

The Arp2/3 complex is a complex of seven proteins and can itself be activated by interaction with proteins containing verprolin central acidic (VCA) domains. VCA domains bind both globular actin and the Arp2/3 complex and are thought to stimulate a conformational change in the quaternary structure of the Arp2/3 complex [Robinson et al., 2001; Goley et al., 2004; Rodal et al., 2005]. In this state the Arp2/3 complex could potentially mimic an actin dimer, overcoming the initial barrier to filament formation and hence create a nucleus supporting spontaneous elongation by addition of further actin monomers. Wiskott-Aldrich Syndrome Protein/WASP family verprolin homologous protein (WASP/WAVE) proteins are characterized by a C-terminal VCA domain, preceded by numerous protein domains postulated to regulate its activity or subcellular localization [Ibarra et al., 2005]. Thus, WASP/WAVE proteins act as nodes through which extracellular signals are converted into the co-ordinated formation of F-actin structures.

There are three mammalian WAVE isoforms. WAVE2 is essential for life and mediates the formation of lamellipodia downstream of Rac in murine embryonic fibroblasts (MEFs) [Yan et al., 2003], as well as regulating the polarization of the Golgi apparatus downstream of MAP kinases [Magdalena et al., 2003; Danson et al., 2007]. The mechanism of WAVE regulation is still not settled, with several models proposed, including regulation through its interaction with a protein complex containing Nap1, Pir, Abi and HSC300 and/or through binding IRSp53 [Ibarra et al., 2005]. It is becoming clear that phosphorylation of WAVE proteins is crucial for their biochemical and cellular function, with both serine and tyrosine phosphorylation playing important roles [Leng et al., 2005; Ardern et al., 2006; Kim et al., 2006; Danson et al., 2007]. We have shown that the activity of WASP is dependent upon phosphorylation of its VCA domain by casein kinase (CK2) [Cory et al., 2003], and others have shown WAVE2 VCA domain phosphorylation by Erk at two sites [Nakanishi et al., 2007]. We sought to characterise WAVE2 VCA domain phosphorylation more fully and report here the identification of five phosphorylation sites in the WAVE2-VCA domain. We found that the previously reported sites were not targets for Erk, but instead that these and three novel sites are CK2 targets. We characterise the varying effects of phosphorylation on the VCA domain's affinity for and ability to activate the Arp2/3 complex. By introducing phospho-deficient mutants into NIH-3T3 cells, we show that phosphorylation of the WAVE2-VCA domain has profound effects on its cellular properties.

## MATERIALS AND METHODS

### Reagents and Cells

NIH-3T3 fibroblasts were grown in DMEM (Invitrogen) supplemented with 10% newborn calf serum (Invitrogen) and pen/ strep. Cos-7 cells were grown with 10% foetal calf serum.

### Antibodies

anti-WAVE2 (D16), anti-GST and secondary HRP linked secondary antibodies were obtained from Santa Cruz Biotech. Inc. Anti-p34Arc was obtained from Upstate Biotech. Inc. Anti-FLAG (M2) was obtained from Sigma. Alexa 568 labelled phalloidin was obtained from Invitrogen.

**Fig. 2:**
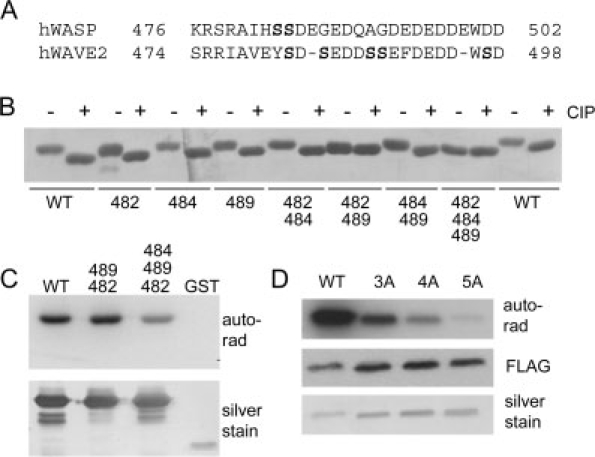
The WAVE2 VCA domain is phosphorylated by CK2. **A:** phospho-peptides used to generate antibodies. pS indicates phosphoserine. **B:** GST-WAVE2 VCA domains were purified from Cos-7 cells and treated with CIP where indicated. Numeric lane descriptors (left panel) indicate residues mutated to alanine, or additive mutants (nA) as described in legend to Fig. 1. Antibodies used for immuno-blotting are indicated on the right of relevant panel. **C:** Endogenous WAVE2 was immuno-precipitated from NIH-3T3 cells and treated with CIP / CK2 as indicated, before immuno-blotting. **D:** WAVE2 VCA domains were purified from Cos-7 cells and treated with CIP / kinase as indicated before immuno-blotting. **E:** Fusion proteins were purified from Cos-7 cells and treated with CIP / kinase as a control for kinase activity.

Phospho-specific antibodies were raised against the phospho-peptides shown in Fig. 2. Peptides corresponding to pser482/4 and pser488/9 were terminated with an amide. Peptides were conjugated to KLH and used to immunize rabbits (Eurogentec Ltd.). Serum from the final bleed was clarified by centrifugation at 25,000g for 30 min, and the supernatant was supplemented with 13 TTBS (0.5 M NaCl, 20 mM Tris pH 8.0, 0.1% Tween- 20) and 1% phosphatase inhibitor cocktail 1 (Sigma p- 2850). This was negatively purified against the nonphospho peptide coupled to epoxy sepharose 6B (ES6B)(GE Healthcare). Flow through was passed over an ES6B phospho-peptide affinity column three times and washed with TTBS until the flow through had an OD280 nm < 0.01. Antibody was then eluted with 0.2 M glycine (pH 2.8) and neutralized with Tris-HCl (pH 8.0).

### Expression Constructs

WAVE2-VCA domain (residues 420–498) was amplified from human cDNA by PCR and cloned into either pEF-Bos downstream of GST or pCMV5 (accession AF239249) downstream of a FLAG epitope. Point mutations were introduced using the Stratagene Quikchange method, and sequenced.

Lentiviral vectors were generated by transfer of EGFP-WAVE2 cDNA into a pSEW sin vector backbone [Danson et al., 2007]. Virus was produced by co-transfection of 293T cells with this plasmid, and pCMVDR8.91 (packaging plasmid) and pMD.G2 (VSV-G pseudotyping plasmid), and recovering cell supernatant after 48 h.

### Recombinant Protein Production

Cos-7 cells (5 × 10^6^) were electroporated with 10 μg of plasmid in electroporation buffer: (120 mM KCl, 10 mM K2PO4/KH2PO4, pH 7.6, 2 mM MgCl_2_, 25 mM Hepes, pH 7.6, 0.5% Ficoll 400) using a Bio-Rad Genepulser at 250 V, 960 lF. After growth overnight COS-7 cells were lysed on ice with lysis buffer:

IGEPAL-630 (1%), 130 mM NaCl, 20 mM Tris- HCl, pH 8.0, 10 mM NaF, 2 mM sodium orthovanadate, 1% aprotinin, 10 μg/ml leupeptin, 1 mM phenylmethylsulfonyl fluoride, 1 mM EDTA.

Lysates were clarificatied by centrifugation and incubated with 50 μl glutathione-Sepharose 4B beads at 4°C for 2 h. Beads were washed three times with LB, twice with 0.8 M LiCl, 20 mM Tris-HCl, pH 8.0, and once with 10 mM Tris-HCl, pH 8.0

### Immunoprecipitation

Endogenous WAVE2 was immunoprecipitated from 3 × 10^6^ NIH-3T3 cells lysed as above. 5 μl Santa- Cruz anti-WAVE2 antibody was incubated per sample for 2 h at 4°C, followed by immobilization with 20 μl Protein A/G sepharose mix, Santa Cruz for a further 2 h. Beads were washed as above.

### Metabolic Labelling

Cos-7 cells were electroporated with GST-VCA or FLAG-VCA domain constructs as described above. After growth ovenight, cells were incubated in growth medium containing 10 Mbq orthophosphate (GE Healthcare) for 3 h, washed and lysed before affinity purification.

### Phosphatase/ Kinase Reactions

GST pull-downs or WAVE2 immunoprecipitates were washed in lysis buffer and 0.8M LiCl, 20mM Tris pH 8.0 prior to incubation with 40 U calf intestinal phosphatase (New England Biolabs) for 30 min at 37°C in the manufacturer's buffer. Following further washing, precipitates were resuspended with 10 U CK2 (New England Biolabs) (in 50 mM Tris pH 7.4, 50 mM NaCl, 10 mM MgCl_2_, 12 μM ATP) or 100 ng recombinant Jnk2 or Erk2 (Upstate Biotech. Inc.) according to manufacturer's instructions, for 45 min at 37°C. These steps were performed prior to elution of the protein where required (see below).

### Affinity Purification

Equal quantities of GST-WAVE2-VCA proteins attached to glutathione sepharose 4B (GS4B) beads were washed thoroughly (0.8 M LiCl) to remove interacting proteins and incubated with fresh lysate from Cos-7 or U937 cells at the indicated concentration, for 2 h prior to washing 33 with lysis buffer.

### Actin Polymerization Assays

GST-WAVE2-VCA domain was over-expressed in Cos-7 cells and purified with glutathione sepharose beads. Where indicated WAVE2 was treated with calf intestinal phosphatase (CIP) then CK2 as described prior to elution three times for 30 min at RT with 200 μl of: 20 mM reduced glutathione, 130 mM NaCl, 50 mM Tris pH 8.0. Eluates were pooled and dialysed against 10 mM Tris pH 8, 1 mM EDTA. Protein concentration was estimated using Bradford reagent with BSA as a standard.

Arp2/3 complex, actin and pyrene actin were from Cytoskeleton Inc. Polymerization assays were performed in 75 mM KCl, 1mM MgCl_2_, 1 mM EGTA, 10 mM imidazole, pH 7.0 with 10 nM Arp2/3 complex and 2.5 μM actin (0.125 μM) pyrene actin), pre exchanged with 200 μM ATP. Fluorescence was measured using excitation of 365 nm and emission at 405 nm.

### Ruffling

NIH-3T3 cells were infected with lentivirus containing cDNA for EGFP-WAVE2 with a multiplicity of infection of one, as calculated using titrations of NIH- 3T3 cells. After 48 h cells were washed and grown on for 1 week prior to freezing. Aliquots were thawed and grown further prior to seeding onto coverslips (coated with poly-L-lysine followed by fibronectin 12.5 lg/ml) in medium containing 0.5% serum at ∼60% confluence. After 24 h cells were stimulated with platelet-derived growth factor (PDGF) ββ (Upstate Biotechnology Inc.) at 1 ng/ml for 25 min then fixed and stained with phalloidin. Cells with one or more F-actin rich ruffle were scored. Data were obtained from three separate experiments each counting 5 or 10 fields per condition.

### Scratch-Wounding

Lentivirally infected cells (see above) were seeded into 4-well glass chambers as described [Danson et al., 2007]. Wounds were generated using a ∼0.5 mm point, and cells tracked as described [Danson et al., 2007].

Leading edges were analyzed after 7.5 h. Movie stills were taken and the ratio between the wound perimeter and the straight line distance calculated to give an indication of raggedness. Data were gathered from three separate experiments with 20 wound edge fields per condition analyzed in total.

### Statistics

Data were analyzed using Graphpad Prism software and Fisher's exact test (ruffling), and ANOVA (persistence and raggedness) as detailed in the figure legends.

## RESULTS

**Fig. 1:**
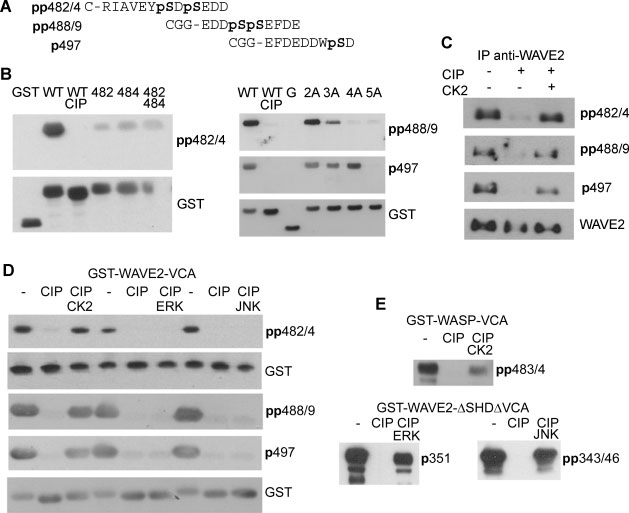
The WAVE2 VCA domain is phosphorylated on multiple serines. **A:** Alignment of the C-termini of human WASP and WAVE2. Bold residues indicate known phosphorylation sites (WASP) and potential sites (WAVE2) in the acidic region. **B:** GST-WAVE2 VCA domains were purified from Cos-7 cell lysates, treated with phosphatase (CIP) as indicated and resolved by SDS-PAGE. WT = wild-type, numbers indicate serine residues mutated to alanine. **C:** GST-WAVE2 VCA domains were metabolically labelled in Cos-7 cells, purified and analyzed by auto-radiography. **D:** FLAG tagged WAVE2 VCA domains were metabolically labelled in Cos-7 cells and analyzed as indicated. 3A = 482A + 484A + 489A; 4A = 3A + 488A; 5A = 4A + 497A.

Phosphorylation of the WASP-VCA domain by CK2 has marked effects on its Arp2/3 complex binding and activation properties [Cory et al., 2003]. We noted that the WAVE2-VCA domain also contains several potential CK2 sites (Fig. 1A) and were interested to know if phosphorylation of this region influenced its actin nucleation activity.

### The WAVE2-VCA Domain Is Phosphorylated on Five Residues

We noted that the GST-tagged WAVE2-VCA domain purified from Cos-7 cells migrated faster on SDS-PAGE following treatment with CIP (Fig. 1B), indicating the presence of one or more phosphates. Three residues within the WAVE2-VCA domain fall within the strictest CK2 consensus sequence of ser/thr-X-X-asp/glu [Kuenzel et al., 1987; Meggio and Pinna, 2003], namely ser482, ser484, and ser489, making them candidates for phosphorylation. Individual mutations of these residues to a non-phosphorylatable alanine did not noticeably alter the mobility of the VCA domain on SDS-PAGE. However, dual mutation of ser482 and ser489 markedly increased the mobility of the VCA domain (Fig. 1B). As the mobility was not increased further by CIP treatment, it appears that phosphorylation of ser482 and ser489 is responsible for the mobility shift. However, as not all phosphorylation events alter protein mobility, we also looked at the incorporation of radiolabelled phosphate into the WAVE2-VCA domain using metabolic labelling. While mutation of ser484 to alanine had no effect on mobility of the WAVE2-VCA domain, it did reduce incorporation of ^32^P when mutated in addition to the dual ser482/489 ala mutation (Fig. 1C), suggesting it is also a phosphorylation site. Additional mutation of the remaining serine residues within the WAVE2-VCA domain—ser488 (4A mutant) and ser497 (5A mutant)—also cumulatively reduced incorporation of phosphate (Fig. 1D), indicating that all five serine residues within the acidic region of the WAVE2-VCA domain are potential phosphorylation sites.

### Endogenous WAVE2 Is Phosphorylated Within the VCA Domain

To determine whether the five serine residues identified above were true phosphorylation targets, we raised antibodies against the appropriate phospho-peptide sequences (Fig. 2A). Due to their close proximity, doubly phosphorylated peptides were used to generate antibodies against p482/p484 and p488/p489. All antibodies recognized wild type GST-WAVE2-VCA domain fusion proteins purified from Cos-7 cells, but had no reactivity against phosphatased forms or GST (Fig. 2B). Mutation of target residues also prevented reactivity, demonstrating that the antibodies are both phospho- and site specific. As expected, the p488/p489 antibody had full reactivity with the dual WAVE2-VCA ser482/ser484ala mutant, but it still retained some reactivity following further mutation of ser489ala in a triple mutant (Fig. 2B, lane 3A). This was lost following further mutation of ser488 ala, demonstrating an absolute requirement for phosphorylation of ser488 and a partial requirement for phosphorylation of ser489 for reactivity.

WAVE2 immunoprecipitates from NIH-3T3 cells were detected by all antibodies (Fig. 2C), showing that WAVE2 is phosphorylated on these sites in vivo (with the caveat regarding ser489 discussed above).

### CK2 and Not MAP Kinases Phosphorylate the WAVE2 VCA Domain

A recent report claimed that WAVE2 ser482 and ser484 are targets for Erk2 in vitro [Nakanishi et al., 2007]. We thought this unlikely (1) due to the lack of an Erk consensus sequence S/T-P and (2) due to the homology of these residues with the CK2 sites in the WASPVCA domain [Cory et al., 2003], a fact overlooked by the authors. We found that dephosphorylated WAVE2 immunoprecipitates could be restored to endogenous levels of phosphorylation by CK2 treatment (Fig. 2C). Next, we purified GST-WAVE2-VCA domain from Cos-7 cells and treated it with CIP. In vitro kinase assays were performed with CK2, Erk2 or Jnk2 and samples were immunoblotted with phospho-specific antibodies. CK2 treatment led to phosphorylation at all five sites, whereas Erk or Jnk treatment did not phosphorylate any (Fig. 2D). Erk and Jnk were shown to be functional by parallel assays demonstrating their ability to phosphorylate sites in the poly-proline rich region of a GSTWAVE2 fusion protein [Danson et al., 2007] (Fig. 2E).

### WAVE2 Is Constitutively Phosphorylated In Vivo

**Fig. 3:**
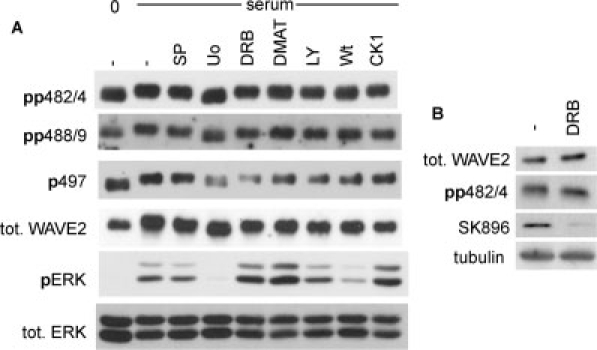
Effect of inhibitors on endogenous WAVE2. NIH-3T3 fibroblasts were starved by plating in 0.5% calf serum and incubating overnight. The indicated inhibitors were added to the culture medium for 3 h prior to stimulation with 10% serum for 7 min where indicated. **A:** Cells were lysed and WAVE2 immunoprecipitated. Samples were split in three and immunoblotted with phospho-specific antibodies. Blots were stripped and reprobed for WAVE2 showing equal loading (the pp488/9 blot reprobe is shown here. The others showed similar loading but are omitted for clarity). Lysates were probed with phospho- Erk antibodies (pERK), stripped and reprobed with antibodies for total Erk (tot. ERK). As A except lysates were blotted for SK896, a phospho-specific antibody recognizing CK2 dependent phosphorylation of N-WASP [Cory et al., 2003]. Tubulin blots were used as a loading control. The top two panels show WAVE2 and pp482/4 immunoblots of immunoprecipitates from cells incubated with DMSO (−) or the CK2 inhibitor DRB, prior to stimulation with serum. − = DMSO (1/1000) vehicle; SP = SP600125 (20 μM) JNK pathway inhibitor; Uo = Uo126 (10 μM) ERK pathway inhibitor; DRB = 5,6- dichloro-1-β-d-ribofuranosylbenzimidazole (50 μM) CK2 inhibitor; DMAT = 2-dimethylamino-4,5,6,7-tetra-bromo- benzimidazole (20 μM) CK2 inhibitor; LY = LY294002 (20 μM) PI3 kinase and CK2 inhibitor; Wt. = wortmannin (100 nM) PI3 kinase inhibitor; D4476 = 4-(4-(2,3-Dihydrobenzo[1,4]dioxin-6-yl)-5-pyridin-2-yl-1H-imidazol- 2-yl)benzamide (50 μM) CK1 inhibitor.

We have used our phospho-specific antibodies to monitor levels of phosphorylation of endogenous WAVE2. We find that WAVE2 is constitutively phosphorylated in the VCA domain in all conditions tested, including serum starvation/ stimulation, growth factor stimulation and scratch wounding among others (data not shown). WAVE2 isolated from NIH-3T3 cells cannot be further phosphorylated on the VCA domain by CK2 treatment (Fig. 2C) suggesting that a large proportion of cellular WAVE2 is phosphorylated in the VCA domain, similar to WASP [Cory et al., 2003], indicating the constitutively active nature of CK2. However, in contrast to WASP we are unable to inhibit this phosphorylation using CK2 inhibitors, despite a reduction of N-WASP phosphorylation at its CK2 dependent VCA domain phosphorylation site (Fig. 3B). A panel of other kinase inhibitors (including PI3 kinase and MAP kinase inhibitors) have been tested under a variety of conditions and have also failed to reduce WAVE2-VCA domain phosphorylation (Fig. 3 and data not shown). The reason for this resistance to CK2 inhibitors is unclear but it may reflect the existence of an inaccessible pool of CK2, a very long-lived and stable phospho-protein species or the involvement of a distinct kinase, resistant to the inhibitors tested. It is also possible that there are spatial, temporal or signal dependent changes in WAVE2 VCA domain phosphorylation that we are unable to detect using our methodology.

### Multiple Phosphorylation Increases the Affinity of the VCA Domain for the Arp2/3 Complex

**Fig. 4:**
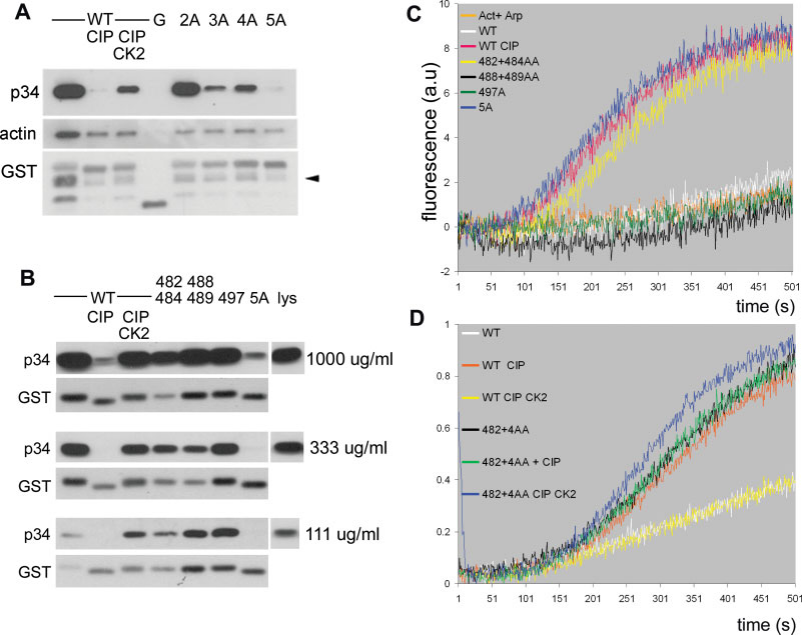
Phosphorylation controls biochemical functions of the WAVE2 VCA domain. **A:** GST WAVE2 VCA domains containing the indicated mutations, were purified from Cos-7 cells. WT VCA domains were treated with phosphatase (CIP) or CIP and CK2 where indicated prior to incubation with fresh U937 cell lysate at 0.7 mg/ml protein. G = GST alone pull-down. Pull-downs were immuno-blotted for p34Arc, actin or GST as shown. Arrow head indicates degradation products. **B:** As A, except double and single alanine mutants were incubated with Cos-7 cell lysate (lys) at the indicated concentrations (**C, D**) Actin polymerization assays performed with GST-WAVE2 VCA domains as indicated.

VCA domain binding to the Arp2/3 complex occurs through residues within the central (C) and acidic (A) regions of the VCA domain [Marchand et al., 2001; Panchal et al., 2003]. Wild type WAVE2-VCA domain purified from Cos-7 cells was highly phosphorylated within the VCA domain (see Fig. 2) and bound the Arp2/ 3 complex from fresh cell lysates (Fig. 4A). Phosphatase treatment of the WAVE2-VCA drastically reduced the amount of Arp2/3 complex bound. The role of phosphorylation was confirmed by the finding that binding could be restored by CK2 treatment (Figs. 4A and 4B). We found in contrast to the findings of Nakanishi et al. that WAVE2-VCA domains bearing a double alanine mutation at sites 482 and 484 showed no reduction in affinity for the Arp2/3 complex (Figs. 4A and 4B). Instead, cumulative mutation of the phosphorylation sites within the VCA domain lead to a decrease in the affinity for the Arp2/3 complex (Fig. 4A). We could not attribute this decrease in binding to phosphorylation of any specific residues as independent alanine substitutions at 482 1 4, 488 1 9, and 497 all bound the Arp2/3 complex with similar affinity over a range of concentrations (Fig. 4B). Binding to G-actin is independent of the phosphorylation state of the VCA domain (Fig. 4A). This would be expected as the G-actin binding V region lies N-terminal to the CK2 phosphorylation sites [Kelly et al., 2006].

### Phosphorylation Inhibits the Activity of the WAVE2-VCA Domain

The effect of phosphorylation on the activity of the WAVE2-VCA domain was tested using in vitro polymerization assays. Wild type (phosphorylated) GSTWAVE2-VCA domains isolated from Cos-7 cells were inactive in these assays, and were unable to stimulate the Arp2/3 complex (Figs. 4C and 4D). Higher concentrations (>100 nM) of WT-WAVE2 VCA were unable to stimulate the Arp2/3 complex (data not shown). However, dephosphorylation resulted in an active VCA domain, capable of stimulating the Arp2/3 complex to nucleate branched actin structures, suggesting an inhibitory role for phosphorylation (Fig. 4C). Use of VCA domain point mutants, revealed that prevention of phosphorylation of ser482 and ser484, but not the other sites, leads to activity akin to the dephosphorylated protein, suggesting that ser482 and ser484 phosphorylation by CK2 is inhibitory. Mutation of ser488 and ser489 together or ser497 alone resulted in an inactive VCA domain (ie comparable to the activity of wild-type VCA domain), showing that their phosphorylation is not inhibitory. Treatment of dephosphorylated wild type VCA domain protein with CK2 caused reversion of the VCA domain to an inactive form, whereas treatment of the ser482/484 ala mutant with CK2 does not suppress its activity (Fig. 4D), confirming that inhibition by CK2 is specific to phosphorylation of these residues. A ser482/484/488/489 ala quadruple mutant in which only ser497 is phosphorylated showed no increase in activity over the 5A mutant (ie. in the absence of inhibitory ser482/484 phosphorylation, data not shown). This shows that the increase in affinity for the Arp2/3 complex in the 4A mutant compared to the 5A (Fig 4A) does not enhance the ability of the VCA domain to stimulate Arp2/3 complex function, in line with the studies divorcing the affinity from the efficacy of Arp2/3 complex activation [Panchal et al., 2003].

### Phosphorylation and Cellular Function of WAVE2

**Fig. 5:**
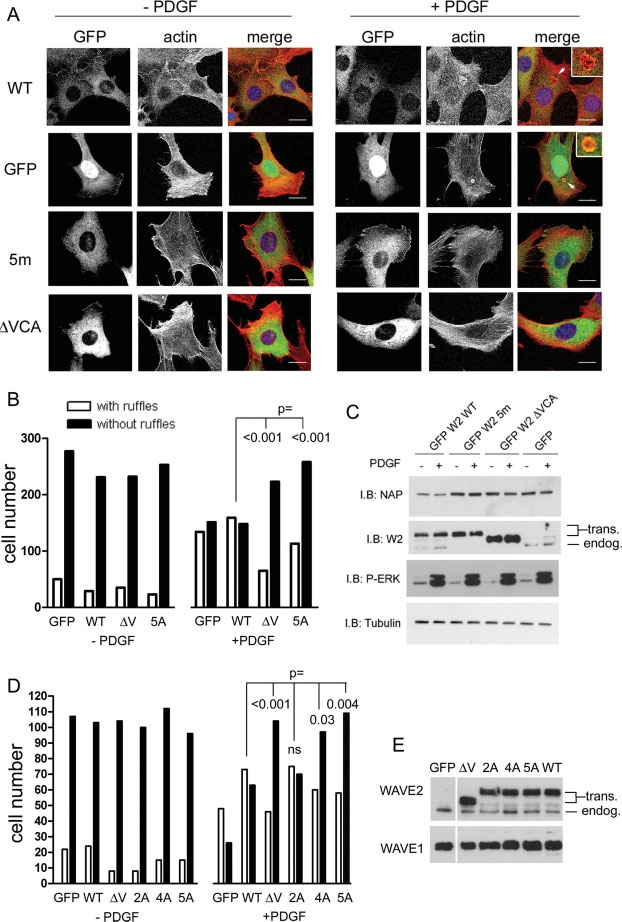
WAVE2 phosphorylation is required for ruffle formation. Phosphorylation of the WAVE2 VCA domain is required for the formation of PDGF-stimulated ruffles. **A:** NIH 3T3 cells expressing GFP-full length WAVE2 transgenes were plated onto fibronectincoated glass coverslips with or without stimulation with PDGF-BB (1 ng/ml) for 25 min prior to fixation with 4% formaldehyde and staining with Alexa568 phalloidin and DAPI. Images are maximum projections of confocal stacks of 10 slices. Scale bars 10 μm. **B:** Quantification of 3 independent ruffling experiments, for each of which, 10 fields were imaged per cell type, per condition (+/− PDGF) ΔV = GFPWAVE2ΔVCA. **C:** Representative immunoblots of PDGF ruffling experiments demonstrating relative transgene expression levels: trans. refers to transgenic WAVE2, endog. to endogenous WAVE2. **D:** As B, except five fields imaged per cell type, 2A = GFP-full length WAVE2 ser482/4AA, 4A = 2A + 488/9AA mutation, 5A = 4A + 497A mutation. **E:** western blot analysis of transgene expression in NIH-3T3 cells used for the analysis in D. Statistics are the results of performing a Fishers exact test using GraphPadPrism software. ns = not significant.

We were interested to know whether VCA domain phosphorylation was required for the cellular function of WAVE2. PDGF stimulation of fibroblasts results in the formation of both peripheral and dorsal (circular) ruffles, a process thought to rely on WAVEs [Suetsugu et al., 2003; Yan et al., 2003]. Imaging of the actin cytoskeleton with fluorescently labelled phalloidin revealed a predominance of circular ruffles that form on the dorsal surface of the cells upon PDGF stimulation. There were also some cells that exhibited small peripheral ruffles. We found that low-level expression of a dominant negative GFP-WAVE2 construct lacking its VCA domain significantly inhibited ruffle formation in response to PDGF (Fig. 5) whereas wild type GFP-WAVE2 or a green fluorescent protein (GFP) control did not. Introduction of GFP-WAVE2 5A also inhibited ruffle formation (Figs. 5A–5C), indicating that phosphorylation of the VCA domain is required for its cellular function, and that in its absence, WAVE2 expression exerts apparently dominant negative effects. We next looked at the effects of cumulatively mutating the VCA domain phosphorylation sites on the effect of WAVE2 transgene expression on ruffle formation. A dual mutation of ser482/4 ala (2A), had no significant effect on ruffle formation, whereas the 4A and 5A mutants did show significant inhibition of ruffle formation in response to PDGF (Figs. 5D and 5E). These findings suggest that the phospho-dependence of the affinity of the WAVE2 VCA domain for the Arp2/3 complex may be important for WAVE2 function in vivo. These data extend the findings of Nakanishi et al, who show that a 2A mutant was adept at restoring ruffle formation in WAVE2 null fibroblasts [Nakanishi et al., 2007]. We find that WAVE1 is upregulated in NIH-3T3 cells following WAVE2 siRNAi treatment (data not shown), making attempts to study the ability of the transgene to rescue cells with knocked-down WAVE2 levels difficult to interpret.

**Fig. 6:**
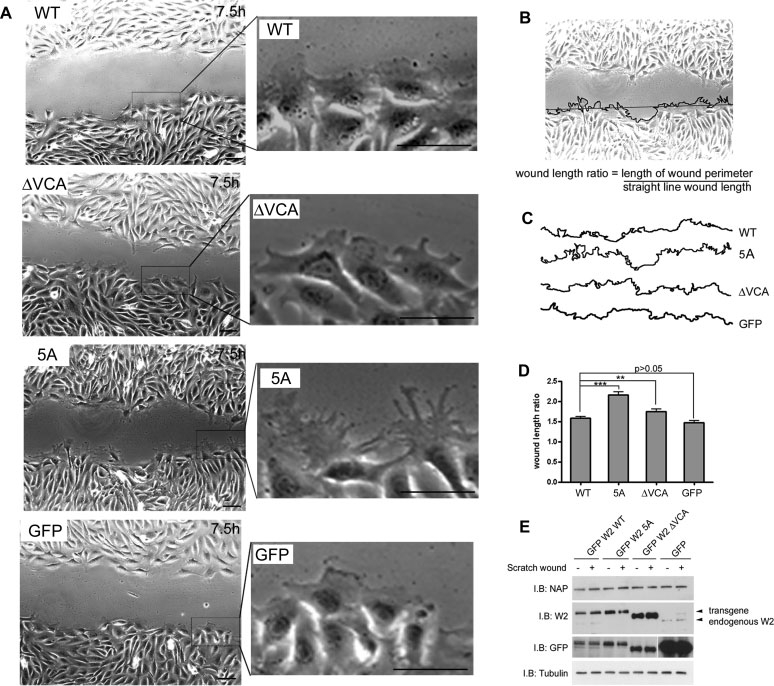
Disruption of leading edge integrity in cells expressing VCA-phospho-deficient WAVE2. **A:** Scratch wound healing assays reveal a requirement for WAVE2 phosphorylation in the maintanence of a intact leading edge. Confluent monolayers of lentivirally-infected NIH 3T3 cells expressing the indicated GFP-WAVE2 transgenes were scratched and phase images taken at 7.5 h. Scale bars 40 μm. **B:** Illustration of the method used to quantify the wound length ratio, the ragged line is the wound perimeter and the straight line the straight line wound lenth. **C:** Representative wound perimeter measurements from all 4 cell types. **D:** Quantification of three separate experiments, *n* = 20 wound edges. *** = *P* < 0.001, ** = *P* < 0.01. Statistics refer to a one-way ANOVA test with Newman-Keuls post test. Error bars represent standard error of the mean. **E:** Expression analysis of confluent monolayers without and without scratching.

**Fig. 7:**
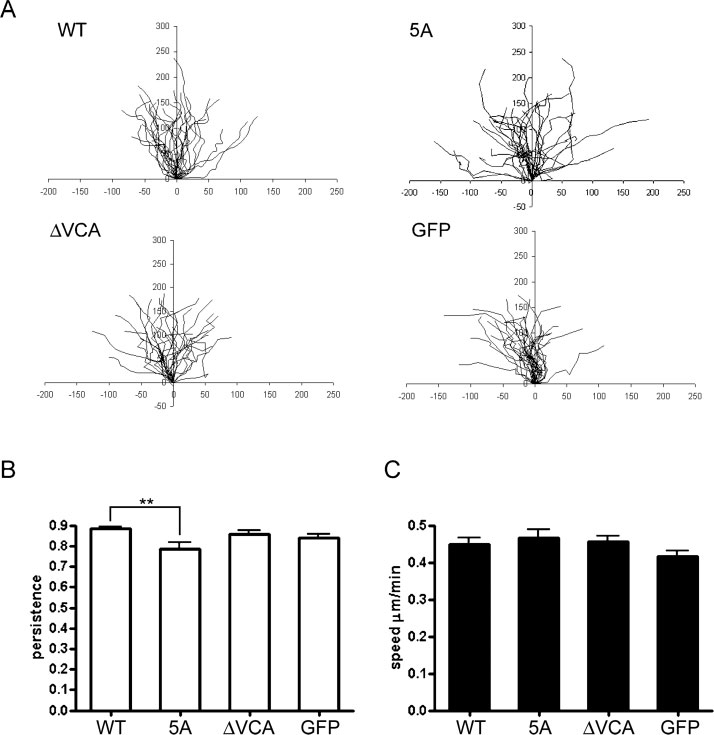
Decrease in migration persistence in cells expressing VCA-phospho-deficient WAVE2. **A:** Traces of cell paths from one representative experiment. Cells expressing the indicated transgenes were grown to confluence and scratch wounds made. Cells at the leading edge of a wound edge were tracked every 30 min for the 6 h preceeding wound closure using ImageJ software. The starting point of each track was normalized to 0 and aligned so the x-axis represents the wound edge. **B:** Persistence of migration calculated by dividing the length of the cell path by the straight-line length. Statistics were performed on data from three replica experiments using GraphPad Prism software to perform a one-way ANOVA test followed by a Newman- Keuls post-test. ** = *P* < 0.01. **C:** Speed of migration obtained from tracking showed no significant difference between conditions. All error bars represent standard error of the mean. In both B and C, a lack of statistical comparisons between datasets indicates a *P* > 0.05.

In order to induce the formation of peripheral cell protrusions (or lamellae) we performed scratch-wounding assays on confluent monolayers of NIH-3T3 cells which respond by extending lamellae into the wound space, followed by migration towards the opposing wound edge. We observed that the wound edge was considerably more ragged in appearance following expression of either delta VCA or 5A WAVE2 (Figs. 6A and 6C). This was quantified by dividing the straight-line distance between the edges of a wound by the total perimeter distance or ‘coastline’ of the wound edge (Fig. 6B). This analysis revealed a significant increase in raggedness caused by WAVE2 5A expression (Fig. 6D) suggesting that the integrity of the leading edge had been compromised and that formation of lamellipodia had been disrupted. Knock-down of WAVE2 has been shown to decrease the persistence of migration direction [Danson et al., 2007], providing another means to test the importance of VCA domain phosphorylation. We observed that low level expression of GFP-WAVE2 5A caused a slight but significant reduction in the persistence of migration (Fig. 7A), with wound edge cells displaying frequent changes in direction compared to GFPWAVE2- WT expressing cells or GFP controls (Fig. 7B). Overall migration speed was not affected (Fig. 7C). These data are consistent with the PDGF ruffling experiments and demonstrate a requirement for phosphorylation of WAVE2 for it to contribute to cell protrusion.

## DISCUSSION

In this paper we show that WAVE2 is subject to multiple functional phosphorylation events within its VCA domain. As the VCA domain is the key output domain of WAVE family proteins, these phosphorylations are well positioned to acutely influence binding affinity for and activation of the Arp2/3 complex.

Two other reports of VCA domain phosphorylation have been published. A recent paper identified ser482 and ser484 as in vitro targets for Erk2, despite the absence of a proline in the 11 position [Nakanishi et al., 2007], generally regarded as the minimal consensus sequence for this kinase [Shaul and Seger, 2007]. Our use of gel shift analysis and phospho-specific antibodies demonstrates a high stoicheometry of phosphorylation by CK2, whereas Nakanishi et al. rely on incorporation of radiolabelled phosphate, which while demonstrating phosphorylation of the VCA domain gives no indication of the stoicheometry. We were unable to show any phosphorylation of the WAVE2 VCA domain using recombinant Erk2, and suggest that the results of Nakanishi et al may reflect a contaminant in their Erk2 preparation or extended incubation times. In addition, the phosphorylated residues we have identified comply largely with established CK2 consensus sequences [Kuenzel et al., 1987; Meggio and Pinna, 2003], and complement an earlier study of phosphorylation of the WASP VCA domain by CK2 [Cory et al., 2003].

In addition to ser482 and ser484 we have used phospho-specific antibodies to identify ser487, ser488, and ser497 as true in vivo phosphorylation sites in WAVE2. Our results suggest that multiple phosphorylation of the WAVE2-VCA domain is required for high affinity binding to the Arp2/3 complex. Abolition of ser482 and ser484 phosphorylation, did not affect the affinity of the interaction when the other phosphorylation sites were occupied, despite the inhibitory effect of phosphorylation of ser482 and 484 on the activity of the VCA domain. The lack of correlation between the affinity of a VCA domain for the Arp2/3 complex and its activity has long been established, suggesting the presence of a two step mechanism [Marchand et al., 2001; Panchal et al., 2003; Beltzner and Pollard, 2008]. Our data on the affinity of the VCA domain for the Arp2/3 complex correlate with work showing that regions of the WAVE1 VCA domain equivalent to ser488 and ser497 form contacts with the Arp2/3 complex whereas ser482 and ser484 do not [Panchal et al., 2003; Kelly et al., 2006]. Hence, phosphorylation of these residues in WAVE2 could potentially affect the binding interface.

In contrast to the findings with WASP we do not detect any effect of VCA domain phosphorylation on the activity of the full length WAVE2 protein in vitro (data not shown). Like WASP, the full length WAVE2 protein is considerably more active than the isolated VCA domain [Cory et al., 2003] and our unpublished data, suggesting that other determinants in the protein can overcome the inhibitory effect caused by phosphorylation of ser482 and ser484 in vitro.

Our cellular experiments show that mutation of the five phosphorylation sites within the WAVE2 A domain generate a protein with properties resembling the DVCA mutant more than the wild-type. In cases such as protrusion raggedness and persistence, the phenotypes are more extreme for the 5A phospho-mutant than the DVCA mutant. This may indicate that the 5A protein is still able to bind actin and sequester it from other, compensatory, nucleation pathways. The 5A phosphomutant VCA domain has a low affinity for the Arp2/3 complex, and while this does not appear to be important for the activity of the isolated VCA domain in vitro, it is possible that a high affinity is required to generate a significant concentration of a WAVE2/Arp2/3 complex in vivo.

The loss of ruffles and the irregular protrusions formed in scratch-wounding assays in the presence of non-phosphorylatable WAVE2, suggest that phosphorylation of the WAVE2 VCA domain is crucial for normal WAVE2 function. In particular the ragged leading edges of scratch-wound cells suggest that the integrity of cell protrusion is compromised. This could potentially be explained by disrupted lamellipodium formation as would be expected in a cell lacking functional WAVE2 [Kunda et al., 2003; Yan et al., 2003]. This may in turn explain the loss of persistent migration, another phenotype observed in WAVE2 depleted cells [Danson et al., 2007].

## CONCLUSIONS

The questions remain as to the nature of the regulation of these phosphorylation events. It appears that phosphorylation is constitutive and not signal dependent, although there may well be stimuli/cellular events that trigger dephosphorylation that we have not yet identified. Bearing in mind the impact of phosphorylations on VCA domain function it is interesting to note the number of sites in various WASP/WAVE family members - WASP/N-WASP have two sites, WAVE2 has five sites only three of which are found in WAVE1 and four of which in WAVE3. Could phosphorylation of the VCA domain be a way of introducing distinct levels of activity and functions of the distinct isoforms?

## Abbreviations

Arp: actin related protein
CIP: calf intestinal phosphatase
CK2: casein kinase 2
F: filamentous
GFP: green fluorescent protein
GS4B: glutathione sepharose 4B
MEF: murine embryonic fibroblast
PDGF: platelet-derived growth factor
VCA: verprolin central acidic
WAVE: WASP (Wiskott-Aldrich Syndrome Protein)-family verprolin homologous protein 2
